# *Bacillus velezensis* MZ09 alleviates DSS-induced colitis in piglets by remodeling the intestinal microbiota activating the SCFAs–GPR43–STAT3 pathway and suppressing NLRP3 inflammasome-mediated pyroptosis

**DOI:** 10.1186/s40104-025-01262-1

**Published:** 2025-08-29

**Authors:** Zhengyi Wang, Xiuyu Fang, Zhihua Yu, Xiangyu Huo, Haiyang Liu, Yongqing Du, Baoming Shi

**Affiliations:** College of Animal Science and Technology, Northeast Agricultural University, Harbin, 150030 P.R. China

**Keywords:** *Bacillus velezensis*, BSCFAs, Disease resistance, GPR43, Piglet, Pyroptosis, STAT3

## Abstract

**Background:**

Inflammatory bowel disease (IBD) is closely associated with intestinal microbiota dysbiosis and metabolic dysfunction. The aim of this study was to explore the protective effects and mechanisms of the probiotic *Bacillus velezensis* MZ09, which produces branched-chain short-chain fatty acids (BSCFAs), against the dextran sulfate sodium (DSS)-induced colitis in piglets.

**Results:**

In this study, a DSS-induced piglet colitis model was established to explore the impact of MZ09. Pretreatment with MZ09 significantly alleviated the symptoms of colitis in piglets. For example, the disease activity index (DAI) score decreased, the length of the colon was restored, and splenomegaly was alleviated. MZ09 enhanced intestinal barrier integrity by upregulating the expression of tight junction proteins such as Claudin-1, Occludin, and ZO-1. Using 16S rRNA analysis, we found that MZ09 could remodel the intestinal microbiota. MZ09 increased the abundance of beneficial bacteria such as Firmicutes and *Lactobacillus* while suppressing the growth of harmful bacteria such as Proteobacteria and *Escherichia-Shigella*. MZ09 also increased the levels of short-chain fatty acids (SCFAs) in the colon. The increased SCFA content activated G-protein-coupled receptor 43 (GPR43), which increased the phosphorylation of signal transducer and activator of transcription 3 (STAT3) and promoted the production of the anti-inflammatory cytokine interleukin-10 (IL-10). Mechanistically, MZ09 mitigated mitochondrial damage via the STAT3/hypoxia-inducible factor 1α (HIF-1α) axis. This action inhibits nucleotide-binding oligomerization domain, leucine-rich repeat and pyrin domain-containing 3 (NLRP3) inflammasome-mediated pyroptosis, thus reducing the release of the proinflammatory cytokines IL-1β and IL-18.

**Conclusions:**

*B. velezensis* MZ09 alleviates DSS-induced colitis in piglets through multiple pathways, including gut microbiota remodeling, SCFAs–GPR43–STAT3 axis activation, and NLRP3 inflammasome-mediated pyroptosis suppression. These findings provide a new theoretical basis for the development of targeted intervention strategies for IBD, suggesting that MZ09 represents a potentially promising therapeutic agent for IBD treatment.

**Graphical Abstract:**

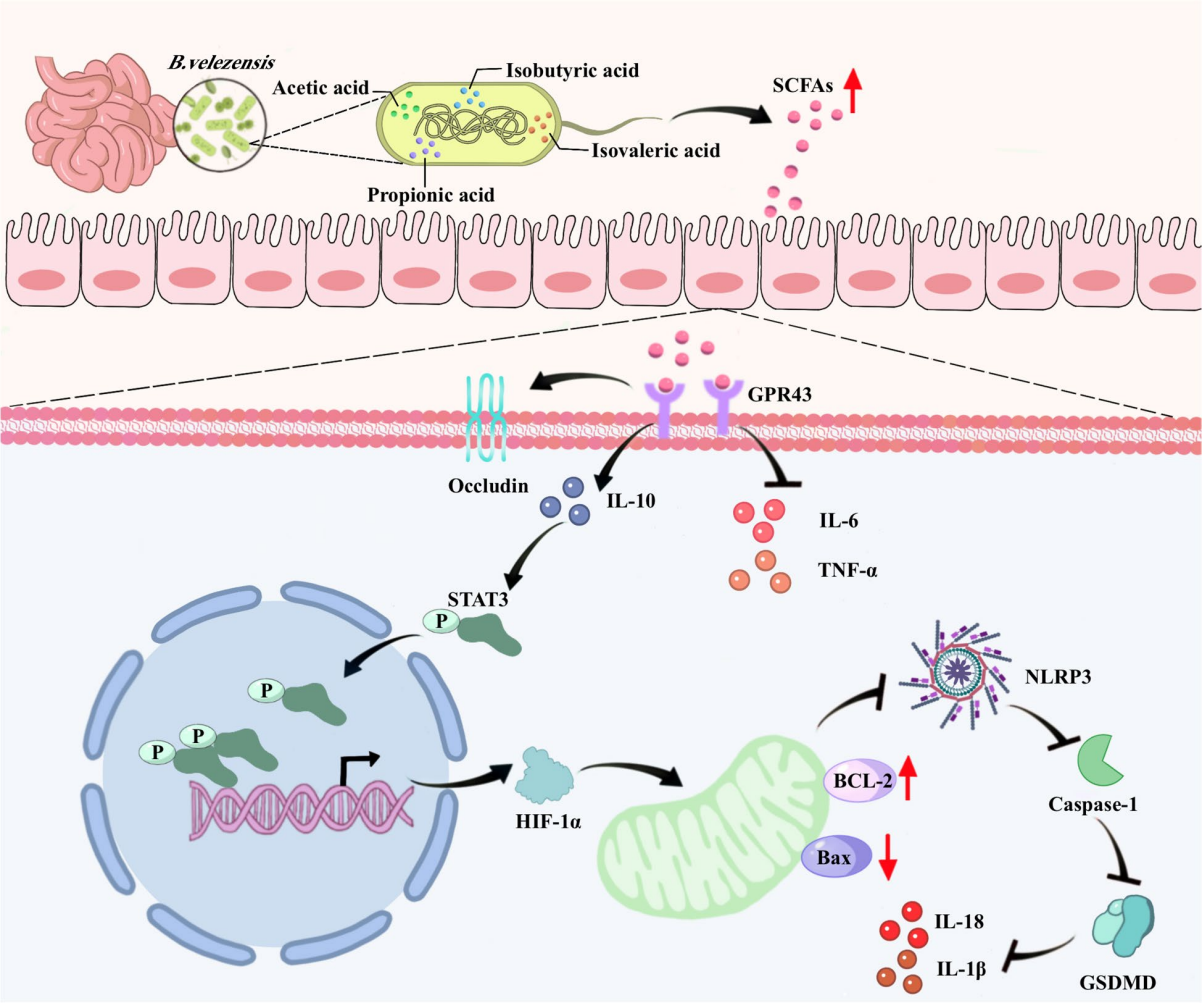

**Supplementary Information:**

The online version contains supplementary material available at 10.1186/s40104-025-01262-1.

## Introduction

Inflammatory bowel disease (IBD) is a heterogeneous disorder. According to the International Classification of Diseases, Crohn’s disease (CD) and ulcerative colitis (UC) are the two main subtypes of IBD [1]. Intestinal inflammation is a crucial health problem in piglet production. During the weaning period, the digestive and immune functions of piglets' intestines are not fully developed, making them extremely prone to colitis [2]. Diarrhea and intestinal inflammation in piglets not only reduce feed efficiency and increase breeding costs but also may lead to the death of piglets, resulting in significant economic losses [3, 4]. Although current research on IBD involves a multifactorial pathogenesis encompassing genetic susceptibility, immune dysregulation, alterations in the gut microbiome, and environmental triggers, the exact interactions of these mechanisms remain incompletely understood [5–7]. Notably, the core driving factor of intestinal inflammation is gut microbiota dysbiosis, which is characterized by the dominance of proinflammatory microorganisms and the depletion of anti-inflammatory species [8]. This microbial dysbiosis affects the body by regulating genes related to IBD susceptibility, influencing the integrity of the intestinal barrier, damaging the immune system, and especially impacting the metabolism of short-chain fatty acids (SCFAs) [9, 10]. Probiotics have been proven to enhance immunity, inhibit infection, and restore microbial balance [11, 12]. Therefore, we screened *Bacillus velezensis*, focusing on alleviating intestinal inflammation in piglets by regulating the gut microbiota and related metabolites.

*Bacillus velezensis*, a novel probiotic that produces SCFAs, has received increasing attention because of its health-promoting properties, such as its antibacterial ability and ability to enhance immune function [13]. *B. velezensis* has recently been approved for inclusion in the "Catalog of Feed Additives" on the basis of sufficient evidence demonstrating its ability to improve intestinal health and enhance animal growth performance. *B. velezensis* presents several unique advantages, making it a highly promising candidate for novel probiotics. First, its ability to form endospores provides excellent environmental tolerance, ensuring high survival rates through the ability of gastric acid and bile salts to reach intestinal inflammatory sites for action [14]. This is a major challenge faced by many nonspore probiotics. Second, the core advantage of *B. velezensis* lies in its strong antibacterial capacity. *B. velezensis* FZ06 inhibits the growth of food spoilage bacteria, and *B. velezensis* QST713 shows broad-spectrum antibacterial activity [15, 16]. In addition, its aerobic/facultative anaerobic characteristics and good production and storage stability also increase the convenience of its practical application. Research has shown that introducing *B. velezensis* can improve the gut microbiota structure, suppress opportunistic pathogens, and augment SCFA-generating bacteria [17]. Our previous study demonstrated that specific aerobic and facultative anaerobic bacteria can effectively prevent DSS-induced colitis in Min pigs. We found that the intestine of Min pigs is rich in *Bacillus* and *Lactobacillus* [18]. Fresh feces from Min pigs were collected to screen for *Bacilli*, from which *B. velezensis* MZ09 was isolated. Its advantages make it an ideal choice for preventing and treating intestinal inflammation in piglets.

SCFAs, which are fatty acids with fewer than six carbon atoms, are the main byproducts of the fermentation of complex carbohydrates such as resistant starch and dietary fiber by the intestinal microbiota [19]. SCFAs enhance the function of the intestinal barrier by promoting epithelial cell growth and repair [20]. Furthermore, by controlling the inflammatory signaling cascade, SCFAs also reduce intestinal inflammation [21, 22]. For example, butyric acid increases the expression of tight junction proteins, including Occludin and Claudin-1, to safeguard the function of the intestinal barrier [23]. Additionally, butyrate stabilizes hypoxia-inducible factor 1 (HIF-1) [24]. HIF-1 improves the hypoxic adaptive capacity of epithelial cells by regulating pathways such as mitochondrial function and glycolysis [25, 26]. Moreover, SCFAs can regulate the immune response and the intestinal barrier through G protein-coupled receptors (GPRs), including GPR41/43 [27]. GPR43 maintains intestinal immune homeostasis and prevents colitis. It achieves this by regulating signaling pathways such as the mTOR/STAT3 pathway. Moreover, GPR43 facilitates the secretion of the anti-inflammatory cytokine IL-10 [28, 29]. Conversely, investigations into isobutyric acid and isovaleric acid remain in their nascent stages. These metabolites mainly originate from the microbial fermentation of branched-chain amino acids, specifically valine, leucine, and isoleucine. Given that their concentrations in the intestine are relatively low, the precise sites of action and the interaction mechanisms with the gut microbiota remain incompletely understood [30].

Regarding the mechanism by which DSS causes intestinal injury, current research has shown that DSS initiates a systemic inflammatory cascade. This occurs through the release of inflammatory factors, impairing the intestinal barrier, and disturbing the microbiota balance. This process leads to the formation of a complex network related to intestinal injury [31, 32]. These mechanisms are interrelated and provide a theoretical basis for establishing a porcine model of intestinal inflammation. We investigated whether *B. velezensis* prevents intestinal injury in piglets with DSS-induced colitis by establishing a piglet colitis model. This study provides a theoretical basis for targeted intervention strategies for IBD.

## Methods

### Culture of bacteria

The initial concentration of *B. velezensis* MZ09 was determined by measuring whether the colony-forming unit (CFU) count of *B. velezensis* MZ09 at different bacterial concentrations, after being cultured on sterile Luria–Bertani (LB) solid medium (Solarbio, Beijing, China) for 24 h, could reach 1 × 10^9^ CFU/mL. *B. velezensis* MZ09 was inoculated into sterile LB broth and cultured at 37 °C under aerobic conditions without light–dark cycle control, with continuous shaking at 120 r/min in an SPH-2102C incubator (ShiPing, Shanghai, China) for 24 h. In order to guarantee the freshness and sufficient provision of the bacterial solution applied in the experiment, all bacteria were prepared in a standardized way, and the cultivation cycle was precisely matched with the experimental schedule.

### Crystal violet biofilm assay

The formation of biofilms was monitored using crystal violet staining. MZ09 was cultured with reference to bacterial culture methods. After inoculation into LB medium, the bacteria were cultivated for an entire night for activation. The bacterial suspension was subsequently diluted to 0.5 McFarland turbidity and diluted 1:100 with new LB medium. One hundred microliters were added to three sterile 96-well polystyrene plates (Corning, New York, USA). After the plates were inoculated and cultured for 24, 48, or 72 h, the culture medium was discarded, and the plates were washed twice with sterile water to remove planktonic bacteria. After 10 min of fixation with 100 μL of methanol, the fixing solution was aspirated and discarded. After the well plate had dried naturally, 200 μL of a 0.1% (w/v) crystal violet solution (Beyotime, Shanghai, China) was added. Then, the staining process was performed at 37 °C for 15 min. Upon removal of the staining agent, the microplate was subjected to three rinses with PBS. Subsequently, it was turned upside-down and allowed to air dry before taking photographs. Next, 125 μL of a 30% acetic acid solution was dispensed into each well. After a 15-min dissociation period at ambient temperature, the staining solution was suctioned back into a fresh well plate. The absorbance was then determined at a wavelength of 570 nm, with 30% acetic acid utilized as the blank reference.

### Bacterial motility

This study included swimming, swarming, sucrose chemotaxis and twitching motility assays to measure bacterial motility [33]. After the strains were activated overnight in LB liquid medium, the bacterial suspension was adjusted to 0.5 McFarland turbidity, diluted at a ratio of 1:100, and then cultured for an additional 24 h. For the swimming assay (at 30 °C), swarming assay (at 37 °C), and sucrose chemotaxis assay (at 37 °C), 2 μL of the bacterial mixture was spotted at the center of the corresponding agar plates of the culture medium. The plates were cultivated at the appropriate temperatures following the full absorption of the bacterial mixture. To assess bacterial motility, the diameter of colony expansion was evaluated 4, 8, 12, and 24 h after inoculation [34]. The twitching motility assay was performed on an LB agar plate. A sterile needle was used to create an inoculation hole at the center of the plate. Subsequently, 2 μL of the bacterial suspension was added, and the plate was incubated at 37 °C for 24 h. Afterward, the agar was removed, and the plate was stained with Coomassie Brilliant Blue (Sigma, Missouri, USA) for 15 min. Afterward, it was rinsed with running tap water until no blue-colored water was removed. Once the samples were dried, images were captured and recorded.

### Animal experimentation

The study utilized 40 castrated male Yorkshire piglets at 21 d of age, selected from a commercial farm for their physiological uniformity [35]. These piglets were separately placed in stainless-steel metabolic cages. Each of these cages was equipped with feed troughs and automatic water outlets. Prior to the official experiment, each piglet had a three-day pre-feeding acclimation period. Following that, the piglets were split into four groups at random based on their body weights, each consisting of ten replicates and one pig. To evaluate the protective effect of *B. velezensis* MZ09 against ulcerative colitis, the specific groupings were as follows: the control group (CON), the dextran sulfate sodium challenge group (DSS), the *B. velezensis* group (MZ09), and the *B. velezensis* prevention group (MZ09 + DSS). Four groups of piglets were fed with an identical diet, and the detailed formulation of the feed is presented in Table S1. The experiment was divided into two stages. The first stage, designated as the prevention period, spanned 21 d, while the second stage, referred to as the challenge period, lasted 5 d. During the 21-day experimental period of the first stage, the MZ09 group and the MZ09 + DSS group received daily oral gavage. 10 mL of *B. velezensis* MZ09 bacterial solution, with a concentration of 1 × 10⁹ CFU/mL, was administered to each piglet in these two groups [36]. The remaining groups underwent treatment with an equivalent volume of sterile physiological saline, delivered through the identical method. The second stage had a duration of 5 d. During this period, piglets were gavaged with DSS (Macklin, Shanghai, China) to establish an enteritis model. The DSS group and MZ09 + DSS group were fasted for 12 h on d 1, and then each piglet was administered at 9:00–11:00 h daily to reduce circadian rhythm effects, tube-fed with 200 mL of 4% DSS solution using a veterinary feeding device (Huihai, Hebei, China). In the next four days, it was changed to 100 mL of 4% DSS solution daily [37, 38]. During this time, the MZ09 group and the MZ09 + DSS group continued to receive treatment with *B. velezensis* MZ09 via the same route as in the first stage, while the CON group continued to receive treatment with sterile physiological saline.

### Sample collection

Upon the conclusion of the experiment, the piglets were humanely euthanized. Immediately after the piglets were euthanized, blood samples were collected from the anterior vena cava, and tissue samples were taken from each individual pig. The blood samples were allowed to stand for a period of 10 min. Subsequently, they were centrifuged at a rotational speed of 3,500 r/min for 10 min. The resulting supernatant, which was the serum, was transferred into sterile centrifuge tubes and stored at a refrigeration temperature of −80 °C. Sterile centrifuge tubes were used to collect the colonic chyme samples. After rinsing with PBS, some of the colonic tissues were cut into circular slices and fixed in 4% paraformaldehyde (Biosharp, Hefei, China). The other parts were cut into 3 mm × 1 mm × 1 mm fragments and submerged in 2.5% glutaraldehyde (Biosharp, Hefei, China) for subsequent detection. For the remaining colon tissues, a small incision was made in the colon segment, and the mucosal surface contents were gently scraped with a sterile spatula and collected into a sterile cryotube. The colon segment was longitudinally opened, and the colon mucosa was scraped off with a glass slide immediately on an ice-cold surface, rinsed with PBS, and collected in a sterile cryotube. All the samples were rapidly frozen in liquid nitrogen and stored at −80 °C before further examination.

### Disease activity index (DAI)

The DAI was determined through the weighted assessment of the percentage of body weight variation, fecal consistency, and fecal occult blood. The scoring guidelines are presented in Table S2. The body weights of all piglets were measured and noted at the identical time daily. The percentage of body weight change was calculated by using the weight registered on the initial day of DSS treatment as a reference. The Fecal Occult Blood Detection Kit (TC0511, Leagene, Beijing, China) was used to identify the fecal occult blood, and the fecal characteristics were assessed every day. All the scorings were performed by the personnel trained in pathology.

### Histological analysis

Once the colonic samples were cut into ring shapes, they were immersed and fixed in 4% paraformaldehyde for a duration exceeding 24 h. Subsequently, these fixed samples were embedded within paraffin blocks. After sectioning, deparaffinization and hydration, they were then stained using H&E staining method. The tissue sections were observed and microscopic photographs were taken using the electronic microscopy imaging system DP73 (Olympus, Tokyo, Japan) at a magnification of ×100 to observe the infiltration of inflammatory cells. Alcian blue staining was used to assess the intestinal mucosal epithelium's secretion in accordance with the manufacturer's instructions (Servicebio, Wuhan, China). To determine the quantity of goblet cells in the colon, the fixed tissues were dehydrated using xylene and absolute ethanol after being stained with Alcian blue for 10–15 min. The goblet cells were enumerated within 10 completely intact crypts that were randomly chosen from each pig's colonic tissue sample.

### Immunofluorescence

After deparaffinization and hydration of the paraffin sections, antigen retrieval treatment was carried out. The primary antibody was applied dropwise after 30 min of blocking with 3% BSA (Servicebio, Wuhan, China), and it was incubated at 4 °C overnight. Subsequently, the slides were washed 3 times in PBS on the decolorizing shaker DS-2S100 (Servicebio, Wuhan, China) with shaking for 5 min each. Appropriate secondary antibodies were then introduced, and the samples were incubated at room temperature in the dark for 50 min. Following this, the tissue sections were treated with DAPI staining solution and kept in the dark for 10 min at room temperature. Following staining, a confocal microscope (Leica, Wetzlar, Germany) was used to get microscopic pictures at a 200-fold magnification. The details including the types, manufacturers, and application ratios of the antibodies that were applied are tabulated in Table S3.

### Transmission electron microscopy

After taking a 1 mm^3^ sample of the colonic tissue, it was initially fixed with 2.5% glutaraldehyde and rinsed three times with PBS. Subsequently, under light-shielded conditions, it was then refixed for a duration of 2 h at room temperature in PBS solution that contained 1% osmium tetroxide (Ted Pella, California, USA). Then, it was washed with PBS and dehydrated through a gradient of ethanol-acetone. After dehydration, the sample was infiltrated with epoxy resin overnight, and embedding was completed by polymerization in an oven at 60 °C for 24 h. Following curing, the embedded block was cut into ultra-thin slices measuring 60–80 nm using an ultramicrotome (Leica UC7). A saturated alcoholic solution containing 2% uranyl acetate (SPI, Pennsylvania, USA) was used to stain the sections in the dark. After de-uranium treatment with 70% alcohol and washing with ultrapure water, they were then rinsed with ultrapure water and gently patted dry with filter paper after being stained once more with a 2.6% lead citrate solution without the presence of carbon dioxide. Images were captured using an electron microscope (HT7800, Hitachi, Tokyo, Japan).

### Enzyme-linked immunosorbent assay (ELISA)

We utilized ELISA kits (Hnybio, Shanghai, China) to measure the concentrations of D-lactic acid (D-LA), diamine oxidase (DAO), and lipopolysaccharide (LPS) in the serum, and carried out quantitative analysis of the target substances through the standard curve. After that, the reaction system was put in a microplate reader, and each well's absorbance values were determined using a 450 nm wavelength. The catalog numbers of ELISA kits are shown in Table S4.

### Microbiota data analysis

The genomic DNA of bacteria was extracted from the intestinal contents using the E.Z.N.A.^®^ Stool DNA Kit. The 16S rRNA gene was amplified using primers with the sequences 5'-GTGYCAGCMGCCGCGGTAA-3' and 5'-GGACTACHVGGGTWTCTAAT-3'. The PCR products were purified using AMPureXP beads. The relevant outcomes were measured using the Qubit system (Invitrogen, California, USA). Standardized sequence counts were used to compute the alpha and beta diversity metrics. Microbial taxonomic group differences were examined using Linear Discriminant Analysis (LDA) Effect Size (LEfSe). The differentially abundant groups within each group were identified using the LEfSe technique after the gut microbiota structure of each group was obtained at the genus or species level.

### Quantification of SCFAs

The gas chromatography method was employed to detect the content of SCFAs in the colonic chyme. First, precisely weigh 2 g of cecal contents and combine them with 2 mL of deionized water. Subsequently, subject the mixture to ultrasonic treatment at a power of 250 W for a duration of 5 min. After that, vortex the mixture to ensure uniform mixing. Following a complete extraction process that lasts for 48 h, centrifuge the mixture at a rotational speed of 12,000 r/min for 10 min. Then, filter the obtained supernatant through a 0.22-µm sterile membrane three times, add a mixture of 5:1 (v/v) metaphosphoric acid and crotonic acid, and shake it thoroughly to mix evenly. Centrifuge and filter the solution again, and finally store it at 4 °C. Use a high-performance gas chromatograph (GC-2010, Shimadzu, Kyoto, Japan) fitted with an HP-INNOwax capillary column (Catalogue number: 19091N-213) to analyze it. Finally, in order to determine the quantity of SCFAs, a standard curve should be established by using standards of SCFAs.

### Western blotting

The proteins within porcine colonic tissues were extracted with RIPA strong lysis buffer supplemented with PMSF and a phosphatase inhibitor. To quantify the protein content, a bicinchoninic acid (BCA) protein assay kit was used. Equal amounts of proteins were separated by sodium dodecyl sulfate‒polyacrylamide gel electrophoresis (SDS‒PAGE) and transferred to polyvinylidene fluoride (PVDF) membranes on the basis of their molecular weights using a wet transblotting system (Bio‒Rad). After blocking with 5% BSA solution at room temperature for 2 h, the membrane was then incubated with primary antibody at 4 °C for 12 h, followed by incubation with a goat anti-rabbit/mouse IgG-HRP secondary antibody (ABclonal, Wuhan, China) diluted 1:1,000 at room temperature for 1 h. After each antibody incubation, the samples were thoroughly washed with TBST. Development was conducted using UVItec gel imaging equipment and the BeyoECL Star Fluorescence Detection Kit. All the aforementioned reagents were procured from Beyotime Biotechnology (Shanghai, China). ImageJ software was used to measure the intensity of the proteins. The details, including the types, manufacturers, and application ratios of the antibodies that were applied, are tabulated in Table S5.

### Statistical analysis

The data analysis mainly involved conducting a two-way ANOVA using SPSS 27.0 to analyze the main effects of MZ09, the main effects of DSS, and the interaction between the two. When the interaction showed significant differences, a one-way ANOVA was performed, and the Duncan's method was used for multiple comparisons. For the analysis of two groups of data sets, an independent samples *t*-test was applied. The data were presented as the mean ± standard error of the mean (SEM), and a *P*-value < 0.05 was used to determine statistical significance. A *P*-value between 0.05 and 0.1 was considered to represent a trend.

## Results

### The characteristics and biological functions of *B. velezensis* MZ09

**Fig. 1:**
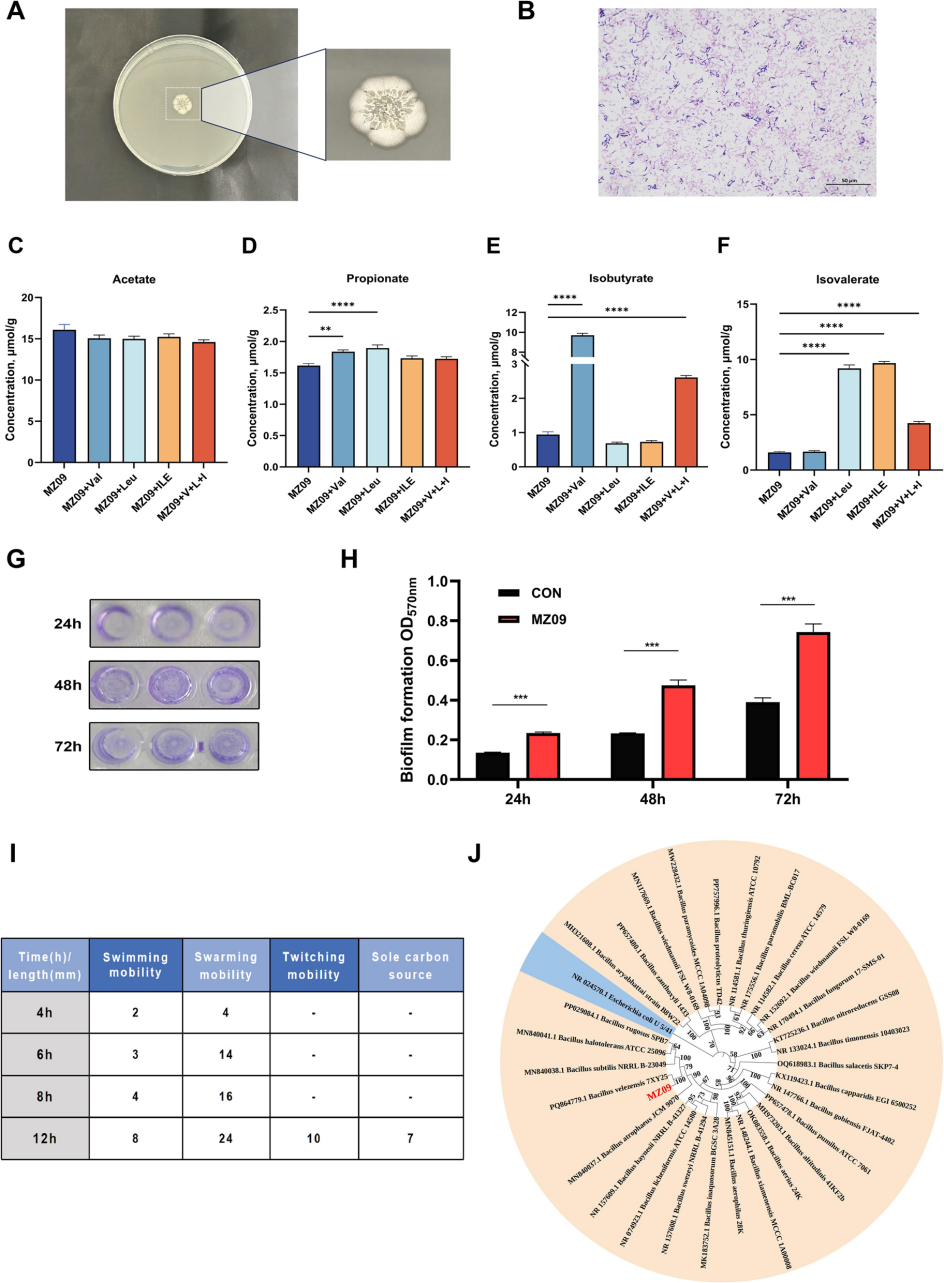
The characteristics and biological functions of *B. velezensis* MZ09. **A** Colony morphology of *B. velezensis* MZ09. **B** Gram-staining results of *B. velezensis* MZ09. **C–F** Changes in the content of SCFAs of *B. velezensis* MZ09 after 48 h culture alone and with the addition of valine, leucine, isoleucine, and an equal-proportion mixture of the three, respectively (*n* = 10). **G**–**H** Biofilm-forming ability of *B. velezensis* MZ09 (*n* = 3). **I** Motility characteristics of *B. velezensis* MZ09. **J** Phylogenetic tree of *B. velezensis* MZ09. Data are presented as the mean ± SEM. ^*^P < 0.05; ^**^P < 0.01; ^***^P < 0.001; ^****^P < 0.0001

In this study, we selected the strain *B. velezensis* MZ09 screened from the intestine of Min pigs, a Chinese native pig breed, as the research subject. It had distinct wrinkles on its surface, and its edges were round or slightly irregular in shape (Fig. 1A). Gram staining showed typical purple rod-shaped cells, which conformed to the characteristics of Gram-positive bacteria (Fig. 1B). The analysis of metabolic characteristics revealed that this strain possesses the capability to produce acetic acid, propionic acid, isobutyric acid, and isovaleric acid. Significantly, the synthesis of BSCFAs was especially remarkable. Hence, this research further assessed the enhancing impact of incorporating branched-chain short-chain amino acids on the biosynthetic process of BSCFAs. The experimental results showed that valine selectively facilitated the synthesis of isobutyric acid, whereas leucine and isoleucine significantly enhanced the yield of isovaleric acid. When the three branched-chain amino acids were added in an equal ratio combination, the total production of BSCFAs exhibited a synergistic effect (*P* < 0.05; Fig. 1C–F). Analysis of the population behavior characteristics of the strain showed that *B. velezensis* MZ09 had the ability to form biofilms, with its bacterial motility primarily characterized by swarming mobility (Fig. 1G–I; Fig. S1). This strain's taxonomic identification as *B. velezensis* was validated by the phylogenetic tree based on the 16S rRNA gene sequence (Fig. 1J).

### *B. velezensis* MZ09 alleviates DSS-induced colitis in piglets

**Fig. 2:**
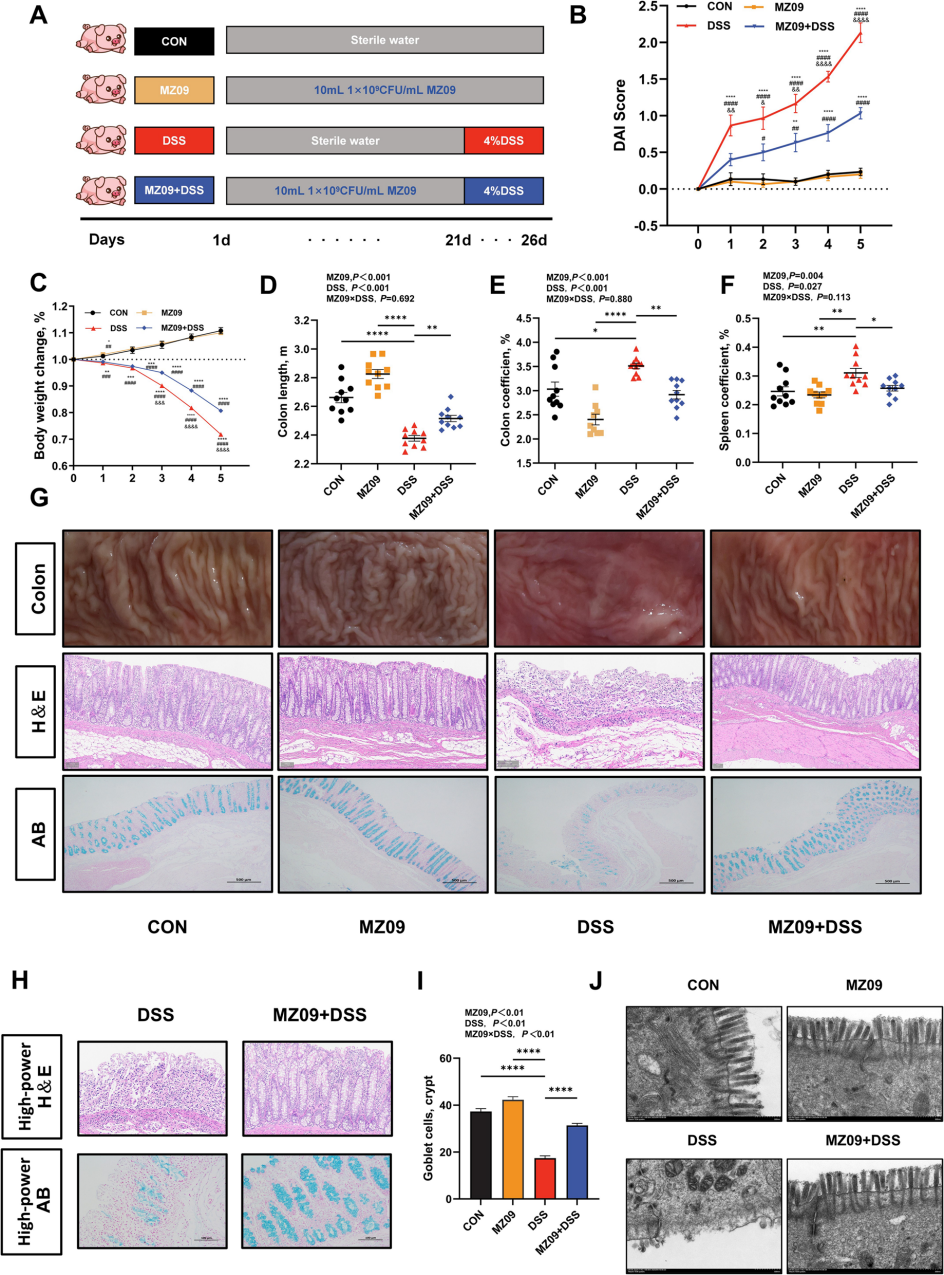
*B. velezensis* MZ09 alleviates DSS-induced colitis in piglets. **A** Twenty-one days before the experiment, the CON group and the DSS group were fed a basal diet, while the MZ09 group and the MZ09 + DSS group were orally administered 10 mL of *B. velezensis* MZ09 bacterial liquid (1 × 10^9^ CFU/mL) daily on the basis of the basal diet. To create a model of colitis, the DSS group and the MZ09 + DSS group were administered intragastric 4% DSS starting on d 22. The other groups were given an equivalent volume of sterile water. A twofold dosage was administered on the first day of the five-day DSS challenge. **B** DAI scores during the DSS challenge period (*n* = 10). **C** A comparison of the groups' body weight change rates (*n* = 10). ^*^P < 0.05, ^**^P < 0.01, ^***^P < 0.001, ^****^P < 0.0001 (vs. the CON group); ^#^*P* < 0.05, ^##^*P* < 0.01, ^###^*P* < 0.001, ^####^*P* < 0.0001 (vs. the MZ09 group); and ^&^*P* < 0.05, ^&&^*P* < 0.01, ^&&&^*P* < 0.001, ^&&&&^*P* < 0.0001 (vs. the MZ09 + DSS group). **D–F** Colon length, colon coefficient, and spleen coefficient comparison (*n* = 10). **G** Representative images of the macroscopic morphology of the colonic mucosa, H&E staining, and AB/PAS staining (*n* = 3). **H** Typical high-power H&E and AB staining images of the DSS group and MZ09 + DSS group (*n* = 3). **I** The number of goblet cells in the colonic tissue (*n* = 10). **J** Typical transmission electron microscopy images of the colonic microvilli. The *P*-value represents the main effect of MZ09, the main effect of DSS, and the interaction between MZ09 and DSS. Data are presented as the mean ± SEM. ^*^P < 0.05; ^**^P < 0.01; ^***^P < 0.001; ^****^P < 0.0001

To investigate the potential therapeutic effect of *B. velezensis* MZ09 on IBD, this study established a colitis model in piglets through the application of 4% DSS [39]. Moreover, the piglets were treated with *B. velezensis* MZ09 (Fig. 2A). DAI analysis indicated that the DSS group had a significantly increased disease score from the initial day of the DSS challenge, and significant differences were noted compared with those of the other three groups (*P* < 0.05; Fig. 2B). On the first day after DSS challenge, the body weight of the DSS group was significantly different from that of the CON group and the group treated with MZ09. The body weights of the DSS group differed significantly from those of the CON group and the MZ09 group on the first day following the DSS challenge, and this difference increased over time (*P* < 0.05; Fig. 2C). Notably, the MZ09 + DSS group did not significantly differ from the CON group until the third day after the DSS challenge, and preintervention with *B. velezensis* MZ09 delayed disease progression. These findings suggest that the colitis model in piglets was successfully established. Moreover, the colon length in the DSS group decreased significantly, whereas the colon length in the MZ09 + DSS group did not significantly differ from that in the other two groups (Fig. 2D). In the MZ09 + DSS group, no significant change was observed, but the colons in the DSS group exhibited marked congestion and edema, which caused their mass to increase substantially compared with those in the other three groups (*P* < 0.05; Fig. 2E). The spleen of the DSS group was distinctly larger and heavier compared with those of the other three groups. On the other hand, following MZ09 intervention, splenomegaly was markedly reduced (*P* < 0.05; Fig. 2F). Furthermore, examination of the morphology of the pigs' colonic mucosa revealed that the colonic mucosa of the pigs in the DSS group was congested and ulcerated, whereas the colonic mucosa of the pigs in the MZ09 + DSS group showed no discernible pathological alterations (Fig. 2G and Fig. S2). The colonic structures in both the CON and MZ09 groups were well preserved as demonstrated by H&E and Alcian blue staining of the colonic tissues. This was evidenced by the presence of an intact submucosal plasma membrane layer and muscular layer, along with the regular and organized arrangement of crypts and mucosal cells. In contrast, the DSS group presented damaged crypt structures in the colonic tissue (Fig. 2G and H; Fig. S3). There was infiltration of inflammatory cells, exfoliation of intestinal epithelial cells, and a reduction in the number of goblet cells. Nevertheless, the application of MZ09 reversed these pathological changes, and the combination of MZ09 and DSS significantly affected the number of goblet cells in the colons of the piglets (*P* < 0.05; Fig. 2I).

### *B. velezensis* MZ09 has a protective effect against intestinal inflammation and barrier dysfunction

**Fig. 3:**
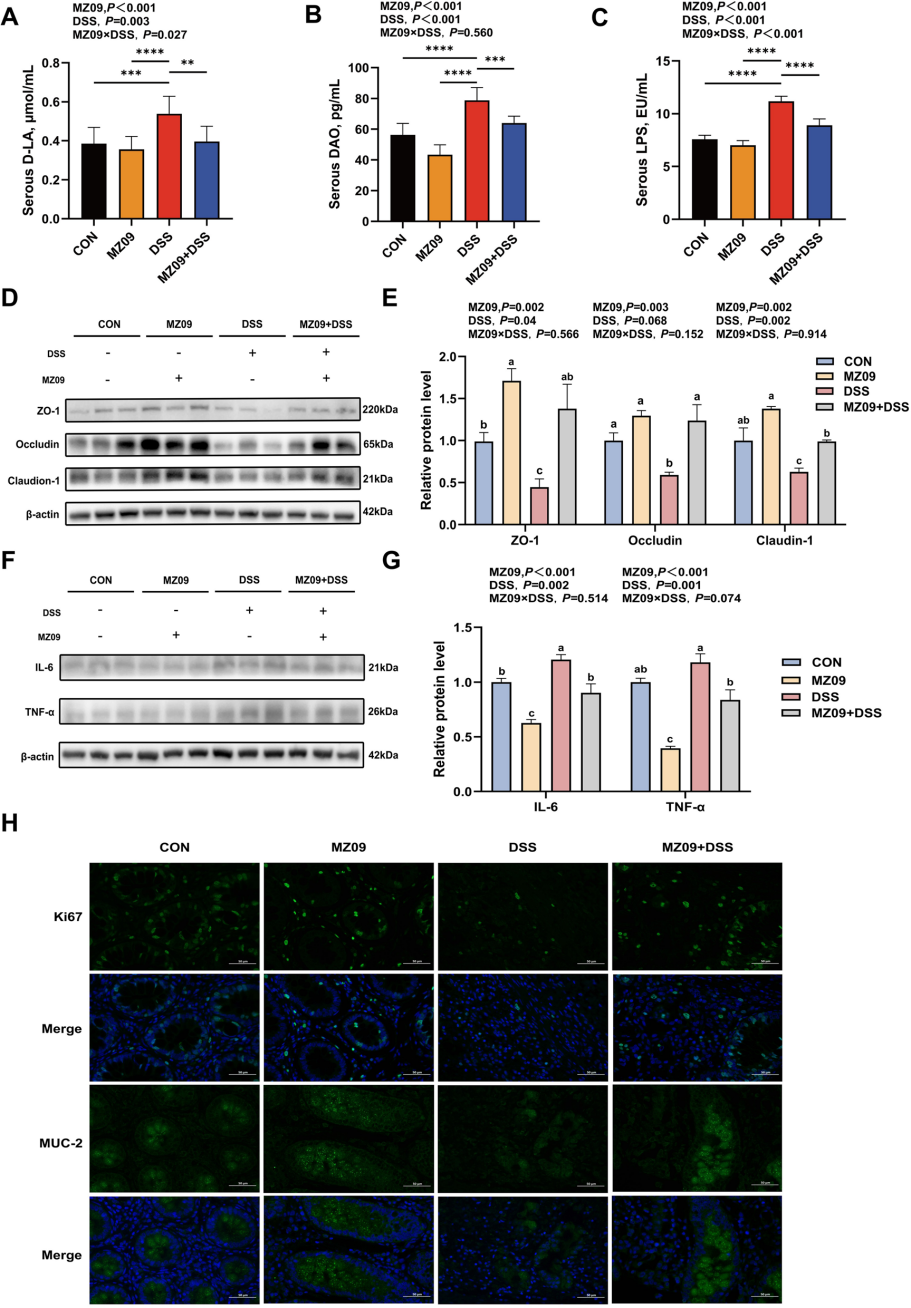
The effects of *B. elezensis* MZ09 on the intestinal barrier and inflammatory factors. **A**–**C** Measurement of D-LA, DAO and LPS in the serum (*n* = 10). **D** and **E** Western blot analysis of the expression of tight junction proteins (ZO-1; Occludin; Claudin-1) in the colonic tissue (*n* = 3). **F**–**G** Western blot analysis of the expression of inflammatory cytokines (IL-6; TNF-α) in the colonic tissue (*n* = 3). **H** Typical fluorescence pictures of colonic tissue showing MUC2, Ki67 (*n* = 3). The *P*-value represents the main effect of MZ09, the main effect of DSS, and the interaction between MZ09 and DSS. Data are presented as the mean ± SEM. ^*^P < 0.05; ^**^P < 0.01; ^***^P < 0.001; ^****^P < 0.0001. ^a–c^Different letters indicate significant differences between groups (*P* < 0.05)

We measured the levels of D-LA, DAO, and LPS in the serum to gauge the colonic damage in piglets in order to analyze the regulatory effects of *B. velezensis* MZ09 on intestinal barrier function and inflammation. The intestinal damage caused by DSS was substantially alleviated by *B. velezensis* MZ09, as evidenced by the significantly increased quantities of these three substances in the DSS group compared with the CON group, MZ09 group, and MZ09 + DSS group (*P* < 0.05; Fig. 3A–C). Moreover, there was a significant interactive effect of feeding MZ09 and challenging with DSS on the levels of D-LA and LPS in the serum of piglets (*P* < 0.05; Fig. 3A–C). The DSS group's expression levels of Claudin-1, Occludin, and ZO-1 were significantly lower than those of the CON group (*P* < 0.05; Fig. 3D and E). However, pre-feeding with *B. velezensis* MZ09 could up-regulate the colonic tight junction proteins to restore the integrity of the intestinal barrier. Additionally, the MZ09 + DSS group significantly decreased the expression levels of TNF-α and IL-6 in the piglets' colons when compared with the DSS group (*P* < 0.05; Fig. 3F and G). Additionally, immunofluorescence analysis showed that the DSS group's MUC2 and Ki67 fluorescence intensity and area were significantly lower than those of the other three groups (Fig. 3H). These results indicate that *B. velezensis* MZ09 alleviated intestinal inflammation by upregulating the expression of tight junction proteins, inhibiting the secretion of pro-inflammatory factors, and restoring the number of goblet cells and the mucus secretion function.

### *B. velezensis* MZ09 alters the intestinal microbiota composition in piglets with colitis

**Fig. 4:**
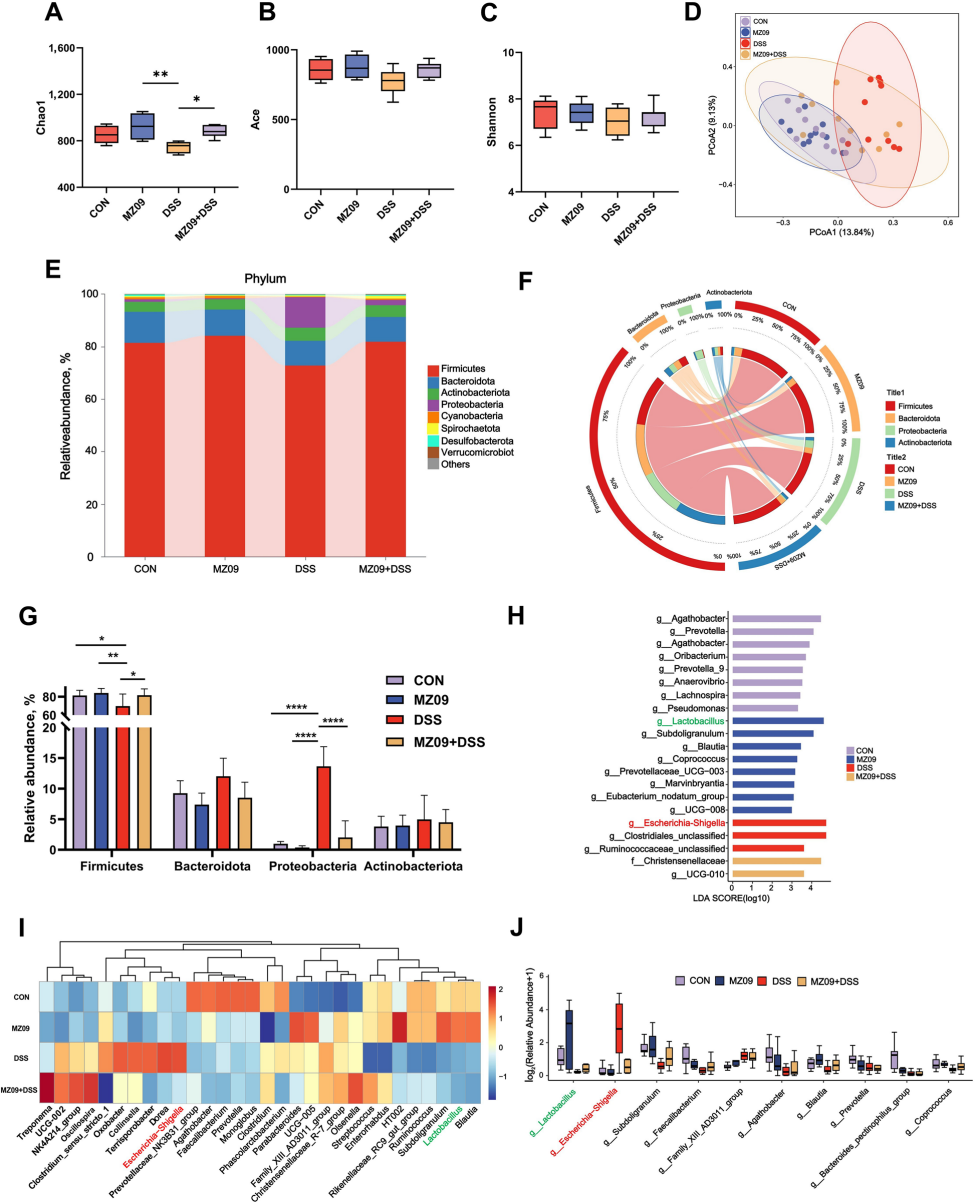
The effect of *B. velezensis* MZ09 on the intestinal microbiota of pigs with colitis induced by DSS. **A**–**C** Comparison of intestinal microbiota α-diversity indices (Chao1, Shannon, and Ace) (*n* = 10). **D** Principal Coordinates Analysis (PCoA) of β-diversity indices (*n* = 10). **E** Composition of colonic microbiota at the phylum level (*n* = 10). **F** Proportional distribution of the four dominant bacterial phyla among groups (*n* = 10). **G** Relative abundances of the four dominant bacterial phyla (*n* = 10). **H** Analysis of differences in microbial abundances by LEfSe (LDA > 3; *n* = 10). **I** Genus-level heatmap showing the top 30 genera with high abundances (*n* = 10). **J** Differences in the abundances of the top ten genera among groups (*n* = 10). Data are presented as the mean ± SEM. ^*^P < 0.05; ^**^P < 0.01; ^***^P < 0.001; ^****^P < 0.0001

We investigated the effects of *B. velezensis* MZ09 on the gut microbiota of pigs with DSS-induced colitis using 16S rRNA gene sequencing. The results revealed that the α diversity index (Chao1) of the DSS group was significantly lower than that of the CON group and that *B. velezensis* MZ09 partially restored the α diversity (*P* < 0.05; Fig. 4A‒C). Principal coordinate analysis (PCoA) indicated that the microbial composition of the DSS group differed significantly from that of the other three groups (*P* < 0.05; Fig. 4D). We also investigated the phylum- and genus-level composition of the gut microbiota and microorganisms associated with colitis. At the phylum level, Firmicutes, Bacteroidetes, Proteobacteria, and Actinobacteria were the main components of the colonic microbiota. Among them, the abundance of Proteobacteria significantly increased in the DSS group, whereas the abundance of Firmicutes significantly decreased (*P* < 0.05; Fig. 4E–G). This phenomenon was effectively reversed in the group fed *B. velezensis* MZ09. Analysis at the genus level revealed that the abundance of *Lactobacillus* in the MZ09 + DSS group was significantly greater than that in the DSS group, whereas the abundance of *Escherichia-Shigella* in the MZ09 + DSS group was significantly lower than that in the DSS group (*P* < 0.05; Fig. 4 H–J). These results indicate that *B. velezensis* MZ09 effectively alleviates the intestinal microbiota dysbiosis caused by DSS.

### *B. velezensis* MZ09 promotes the expression of GPRs by increasing the production of SCFAs

**Fig. 5:**
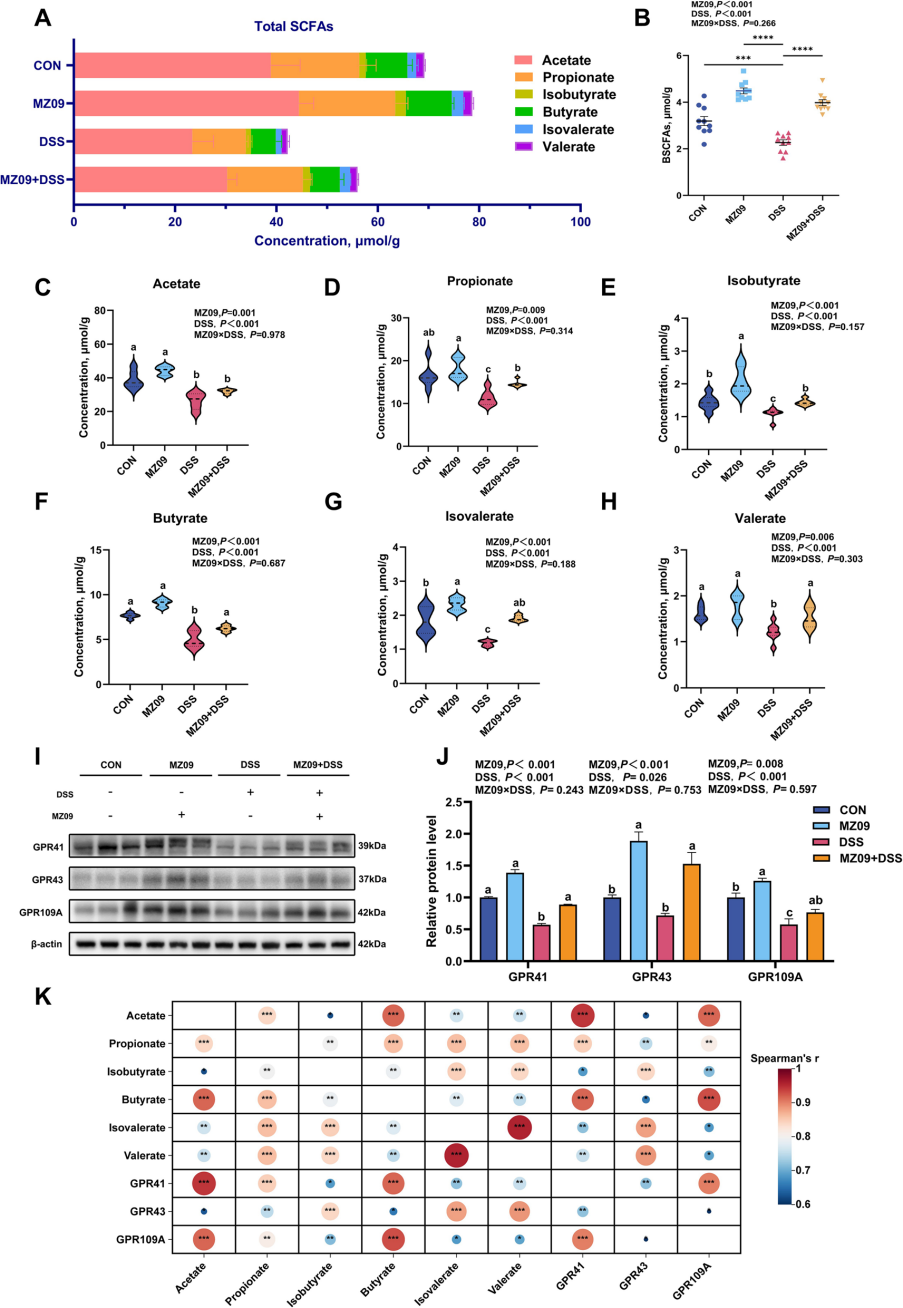
Effect of *B. velezensis* MZ09 on the SCFAs–GPRs signaling pathway in piglets with IBD. **A** The colon's total SCFAs content (*n* = 10). **B** Total BSCFAs content in the colon (*n* = 10). **C**–**H** Concentrations of SCFAs in colonic contents (acetate; propionate; butyrate; isobutyrate; valerate; isovalerate) (*n* = 10). **I** and **J** Western blot analysis of GPR41, GPR43, and GPR109A expression in colonic tissues (*n* = 3). **K** The Spearman correlation between SCFAs and GPRs. Circle size and color intensity reflect the magnitude of Spearman’s correlation coefficients, with larger/redder circles denoting stronger correlations and smaller/bluer circles weaker ones. The *P*-value represents the main effect of MZ09, the main effect of DSS, and the interaction between MZ09 and DSS. Data are presented as the mean ± SEM. ^*^P < 0.05; ^**^P < 0.01; ^***^P < 0.001; ^****^P < 0.0001. ^a–c^Different letters indicate the significant difference between groups (*P* < 0.05)

SCFAs alleviate intestinal inflammation by inhibiting proinflammatory cytokines and serve as crucial metabolites in the protection of the colon. This study demonstrated that *B. velezensis* MZ09 not only metabolizes and produces SCFAs but also significantly increases the abundance of SCFA-producing bacteria in the gut microbiota. We examined the amount of SCFAs in the colonic contents to determine how *B. velezensis* MZ09 affects the levels of SCFAs as microbial metabolites in the gut of patients with colitis. The results revealed that *B. velezensis* MZ09 considerably increased SCFA content in the colon, whereas the DSS group presented significantly lower quantities of total SCFAs, BCFAs, and six main SCFAs, including acetic acid (*P* < 0.05; Fig. 5A–H). Research has demonstrated that G protein-coupled receptors are capable of identifying SCFAs. Using Western blotting, we investigated the expression of these receptors in the colonic tissues. GPR41, GPR43, and GPR109A protein expression levels in the DSS group were significantly lower than those in the CON group (*P* < 0.05; Fig. 5I and J), whereas *B. velezensis* MZ09 significantly increased their expression, especially that of GPR43. We further explored the relationships between SCFAs and GPRs using Spearman correlation analysis. As shown in Fig. 5K, acetate predominantly activated GPR41. In addition, isovalerate played a major role in GPR43, and butyrate demonstrated primary activity at GPR109A. This result suggested that, by improving the composition and structure of the gut microbiota, *B. velezensis* MZ09 controls the levels of SCFAs as microbial metabolites. Consequently, it activates the SCFA receptor (GPR43) in the colon and exerts an anti-inflammatory effect.

### Effects of prefeeding with *B. velezensis* MZ09 on pyroptosis in cells from piglets with DSS-induced colitis via the IL-10/STAT3/HIF-1α signaling pathway

**Fig. 6:**
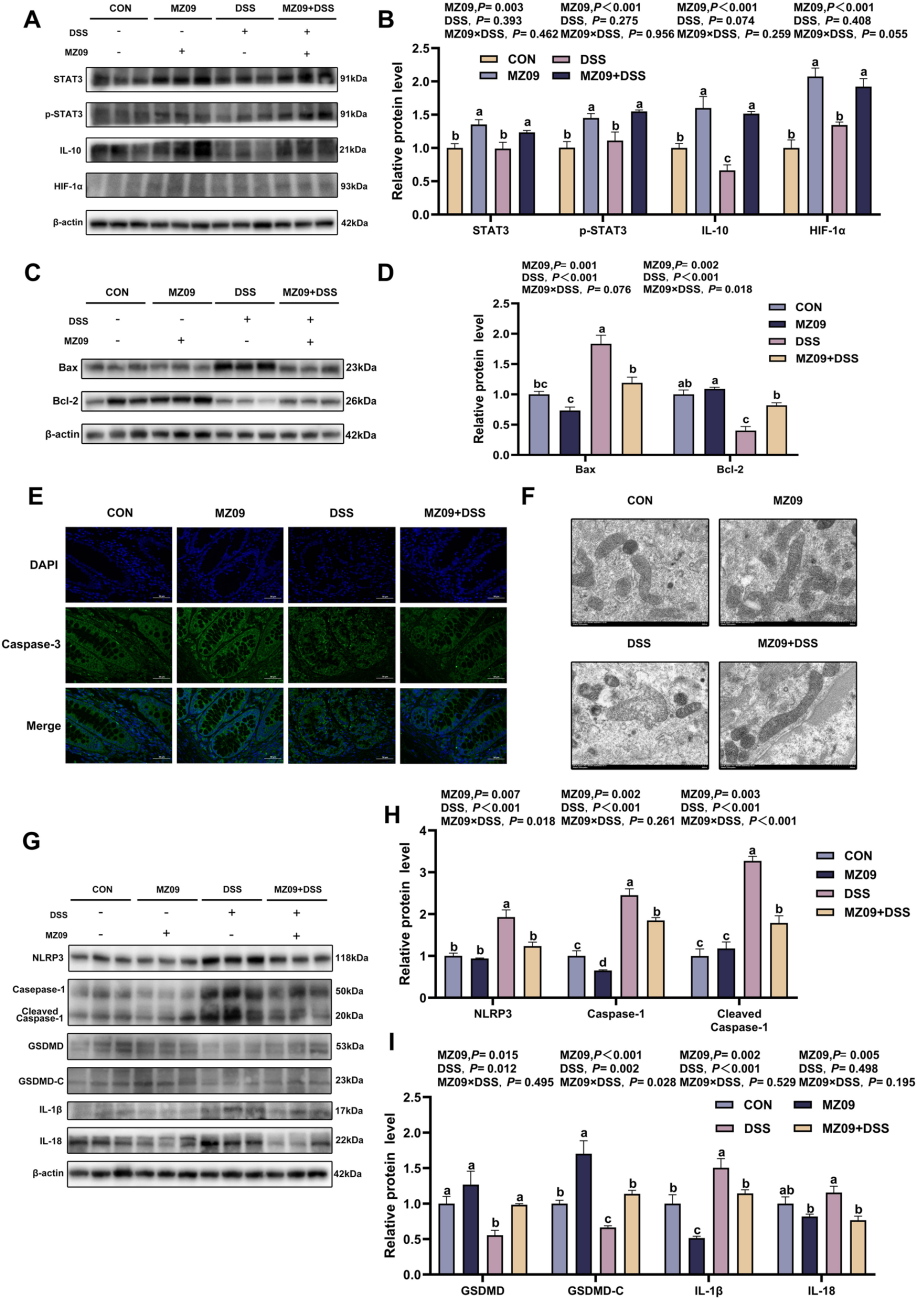
Prefeeding with *B. velezensis* MZ09 alleviates mitochondrial damage and pyroptosis by activating the STAT3 signaling pathway, thereby relieving colitis. **A **and **B** Western blot analysis was used to determine the protein expression levels of STAT3, p-STAT3, IL-10, and HIF-1α in the colon tissues (*n* = 3). **C **and **D** Analysis of the protein expressions of Bax and Bcl-2 in the colon tissues (*n* = 3). **E** Representative images of immunofluorescence staining of Caspase-3 in the colon tissues (*n* = 3). **F** Transmission electron microscopy images of the ultrastructure of mitochondria in colon cells (*n* = 3). **G**–**I** Western blot was used to detect the expressions of proteins related to pyroptosis: NLRP3, Caspase-1 and its cleaved form (Cleaved Caspase-1), full-length GSDMD and its C-terminal (GSDMD-C), IL-1β and IL-18 (*n* = 3). The *P*-value represents the main effect of MZ09, the main effect of DSS, and the interaction between MZ09 and DSS. Data are presented as the mean ± SEM. ^a–d^Different letters indicate the significant difference between groups (*P* < 0.05)

To verify whether *B. velezensis* MZ09 alleviates colitis in piglets through GPR43-mediated STAT3 expression, we detected STAT3 phosphorylation levels. Compared with those in the DSS group, the STAT3 and p-STAT3 protein expression levels in the MZ09 + DSS group were significantly greater (*P* < 0.05; Fig. 6A and B), which might indicate that *B. velezensis* MZ09 is involved in the regulation of inflammation by modulating the STAT3-related pathway. We then measured IL-10 protein expression levels in the colonic mucosa, considering that IL-10 is an upstream regulatory molecule in the STAT3 signaling pathway. The results revealed that the IL-10 protein level in the MZ09 + DSS group was significantly greater than that in the DSS group (*P* < 0.05; Fig. 6A and B). Moreover, the HIF-1α protein, a downstream effector of STAT3, was also significantly increased in the MZ09 + DSS group (*P* < 0.05; Fig. 6A and B), confirming STAT3 activation in the colonic tissue. Considering the antiapoptotic property of STAT3 and the role of HIF-1α in mediating the hypoxic protection pathway and participating in glycolysis to support cell proliferation, we found that the apoptosis-related proteins Bax and Bcl-2 were expressed. *B. velezensis* MZ09 prevents colonic cell apoptosis (*P* < 0.05; Fig. 6C and D). A significant interactive effect of MZ09 feeding and DSS challenge on Bcl-2 expression was observed (*P* < 0.05; Fig. 6C and D). Apoptosis is mediated by the overexpression of the Bax protein, which is found upstream of the mitochondria and regulates the activation of the downstream Caspase-3 protease. Immunofluorescence revealed that the caspase-3 fluorescence intensity and area were greater in the DSS group compared with other three groups (Fig. 6E). Further observation using electron microscopy revealed that DSS treatment disrupted the mitochondrial cristae morphology, leading to mitochondrial damage (Fig. 6F). We hypothesized that *B. velezensis* MZ09 promotes STAT3 phosphorylation to reduce ROS production and maintain mitochondrial permeability. The NLRP3/Caspase-1/GSDMD pathway is a crucial mechanism for pyroptosis, and mitochondria are crucial for the control of the NLRP3 inflammasome. The protein expression levels of NLRP3, cleaved caspase-1 and IL-1β in the DSS group were significantly greater than those in the CON group (*P* < 0.05; Fig. 6G–I), indicating that pyroptosis was promoted. MZ09 and DSS had a significant interactive effect on NLRP3 and cleaved caspase-1 expression (*P* < 0.05; Fig. 6G–I). In contrast, C-GSDMD expression in the MZ09 + DSS group was significantly greater than that in the DSS group. Additionally, MZ09 feeding and challenge with DSS had a significant interactive effect on C-GSDMD (*P* < 0.05). Moreover, it inhibited GSDMD by caspase-1 and reduced the concentrations of the proinflammatory cytokines IL-1β and IL-18 (*P* < 0.05; Fig. 6G–I). These findings suggest that *B. velezensis* MZ09 inhibits NLRP3 inflammasome activation, interferes with pyroptosis, inhibits proinflammatory cytokine production, and ultimately reduces inflammation.

## Discussion

Given the pivotal role of intestinal inflammatory injury in the pathological process of IBD, researching preventive functional substances and understanding their mechanisms are crucial. *B. velezensis* has garnered significant attention for its extensive applications in agriculture and plant-related fields; however, research in livestock and poultry remains limited [40]. We investigated various characteristics of MZ09 to determine its physiological functions as a feed-based probiotic and leveraged its acid-producing ability as an intervention strategy to alleviate intestinal inflammation in piglets. Our team previously reported that BSCFAs improve intestinal homeostasis and alleviate colitis [39, 41]. Therefore, we further investigated the protective effect of the BSCFA-producing probiotic *B. velezensis* MZ09 on the colon in a DSS-induced piglet colitis model. In this study, we demonstrated that *B. velezensis* MZ09 alleviates intestinal inflammation by reshaping the composition of the intestinal microbiota and enhancing intestinal barrier function, providing a new theoretical basis for its application in the prevention and treatment of IBD.

BSCFAs prevent intestinal inflammation by repairing the intestinal barrier, improving intestinal permeability, and activating GPRs to regulate the immune and metabolic functions of the host. Therefore, strains capable of generating BSCFAs have emerged as effective options for addressing intestinal disorders. Notably, *B. velezensis* MZ09 has the capacity to synthesize SCFAs, a trait typical of traditional probiotics. Moreover, it remarkably increases the biosynthesis efficiency of BSCFAs via a distinctive metabolic route involving branched-chain amino acids such as valine and leucine. This specificity may constitute its core competitive advantage, which sets it apart from other strains. The intestinal colonization efficiency of probiotics is related to their biofilm formation ability, and this characteristic is a key functional indicator of the strain's adaptation to the host environment [42]. Strains with the ability to form biofilms independently can increase their tolerance to the intestinal environment, thus gaining an advantage in terms of niche occupation [43, 44]. *B. velezensis* MZ09 primarily secretes surfactants through swarming motility, thereby adhering to the intestinal epithelium, effectively resisting the acidic intestinal environment, and exhibiting greater survival ability than pathogenic microorganisms [45, 46]. Probiotics require a comprehensive assessment of their susceptibility to antimicrobial agents, serving as a foundational measure to ensure their safety and efficacy in animal feed applications [47]. We previously used antibiotics with MIC criteria to conduct in vitro antibiotic susceptibility testing on *B. velezensis* MZ09. We found that MZ09 was sensitive to all the antibiotics specified in the criteria and exhibited strong antibiotic susceptibility. These results support the promising prospect of *B. velezensis* MZ09 as a safe and efficient feed probiotic.

Many studies have shown that when probiotics and SCFAs are administered, they often lead to an improvement in colitis symptoms [48]. Therefore, to thoroughly assess the possible impacts of *B. velezensis* MZ09, a DSS-induced colitis model was created in piglets for this study. Based on assessment of the DAI, the effective establishment of the DSS-induced colitis model was confirmed. The group challenged with DSS presented characteristic clinical symptoms such as a decrease in body weight, loose stools or diarrhea, and the presence of bloody stools. These findings provide evidence that the colitis model was successfully created and that DSS indeed elicited the expected pathological effects. Our study revealed that preadministering *B. velezensis* MZ09 not only delayed the onset of symptoms but also reduced DAI severity. Further analysis revealed that this strain alleviates the pathological characteristics of weight loss, colon shortening, and splenomegaly in piglets with colitis. The spleen and the colon are important components of the immune system [49]. The spleen plays important roles in the body's immunity, including antigen processing and antibody production [50]. In contrast to other tissues, the colonic mucosa harbors a substantial number of lymphocytes. These cells play critical roles in preserving tissue homeostasis and controlling the local immune response [51]. Changes in the spleen index (spleen mass/body weight) and the colon index (colon mass/body weight) reflect the state of immune function [52]. These results reflected the potential of *B. velezensis* MZ09 to regulate the metabolic load of immune organs and maintain immune homeostasis in piglets. During the collection of colonic samples, we systematically documented the macroscopic morphological characteristics of the colonic mucosa, encompassing mucosal coloration, vascular texture, and the presence of pathological alterations, such as erosions and ulcers. Pathology revealed clear erosion and necrosis of the colonic mucosal epithelium, reduced crypts, loss of goblet cells, thickened muscularis mucosae, and widespread inflammatory cell infiltration. These findings collectively suggest that DSS treatment leads to destruction of the integrity of the tissue structure and an increase in the histopathological score. These findings confirmed that *B. velezensis* MZ09 has a protective effect on colitis-related tissue damage.

In cases of IBD, tissue injury causes an increase in serum D-LA and DAO levels. This subsequently leads to an increase in intestinal permeability and compromised integrity of the intestinal barrier [53, 54]. Simultaneously, it also upsets the equilibrium of the intestinal microbiota, which increases LPS blood levels and accelerates IBD progression [55]. However, *B. velezensis* MZ09 regulated the abovementioned pathological processes to reduce the damage caused by colitis to the intestinal barrier. IBD induces intestinal barrier dysfunction by reducing the expression of TJ proteins (Claudin-1, Occludin, and ZO-1) in colonic tissues, which consequently increases susceptibility to colitis [56, 57]. We tested the effects of *B. velezensis* MZ09 on the intestinal TJs of piglets with IBD induced by DSS. *B. velezensis* MZ09 positively influences the expression of TJ proteins in colonic tissues. Damage to these tight junctions can trigger an excessive immune response and cytokine release. Simultaneously, an overabundance of proinflammatory cytokines triggers apoptosis and rupture of the intestinal epithelial barrier as well as intestinal epithelial cells, which results in sustained inflammation and harm to the colon [58]. Our results showed that prefeeding with *B. velezensis* MZ09 reduces the levels of the proinflammatory cytokines IL-6 and TNF-α in a DSS-induced colitis model, thus alleviating colonic damage. Additionally, a crucial part of the intestinal barrier is the mucus layer [59]. In addition to serving as a lubricating layer on epithelial cells, the mucus barrier stabilizes the microbial community and offers a favorable environment for bacterial colonization [60]. *B. velezensis* MZ09 significantly increased MUC-2 levels in the colonic tissues of DSS-induced piglets, facilitating repair of the intestinal mucus barrier.

Dysbiosis and translocation of the gut microbiota are common pathogenic characteristics of IBD [61, 62]. In this study, microbial diversity analysis confirmed that DSS-induced colitis markedly perturbed the gut microbiota composition in piglets. Conversely, intervention with *B. velezensis* MZ09 efficiently increased microbial abundance and restored microbial diversity. In the DSS model, the typical gut microbiota, dominated by Firmicutes and Bacteroidetes, experienced significant compositional changes. In particular, unusual increases in Proteobacteria proliferation and decreases in Firmicutes are noted [63]. Notably, prefeeding with *B. velezensis* MZ09 significantly increased the relative abundance of Firmicutes in the gut of DSS-treated piglets. It also curbed the overproliferation of Proteobacteria. As a crucial component of Firmicutes, *Lactobacillus* performs probiotic functions. It bolsters intestinal barrier function, suppresses pathogenic bacteria, and improves the microecological environment [64, 65]. A high abundance of Proteobacteria is a sign of microbiota dysbiosis. The abnormal proliferation of *Escherichia-Shigella* in the gut can cause toxic mediators, such as LPS, to easily enter the body and trigger an inflammatory response [66]. *B. velezensis* MZ09 significantly increased *Lactobacillus* levels in the gut but significantly decreased the abundance of *Escherichia-Shigella*, thereby mitigating the dysbiosis of the gut microbiota induced by DSS. The metabolites of the gut microbiota are important indicators of the host–microbe relationship and are essential for preserving immunomodulation and gut homeostasis [67]. Metabolites generated by the gut microbiota play a fundamental role in maintaining gut homeostasis and regulating the immune response. They act as key mediators in the connection between the host and microorganisms. Studies have shown that the gut microbiota can reshape the gut microecological environment and maintain gut homeostasis through SCFA synthesis [68]. We found that *B. velezensis* MZ09 optimized the metabolic environment for SCFAs by reshaping the intestinal microbiota, increasing the abundance of SCFA-producing bacteria (such as *Lactobacillus* and *Christensenella*), and inhibiting pathogens. These findings indicate that MZ09 enhances SCFA biosynthesis by remodeling the microbiota. Additionally, *B. velezensis* MZ09 protected intestinal microbial diversity and promoted the growth of SCFA-producing bacteria by regulating the gut flora, thereby increasing the levels of SCFAs.

SCFAs, which are significant products of gut microbiota metabolism, are vital for maintaining gut homeostasis [69]. In this study, prefeeding with *B. velezensis* MZ09 significantly elevated the levels of SCFAs in the colon, particularly those of BSCFAs. This finding aligns with earlier results of this study, indicating that *B. velezensis* MZ09 potentially metabolizes host branched-chain amino acids to increase the biosynthesis of BSCFAs. Research has demonstrated that the three key G protein-coupled receptors for SCFAs are GPR41, GPR43, and GPR109A [70]. The SCFAs–GPRs signaling pathway is crucial for maintaining intestinal barrier stability. SCFAs improve intestinal barrier function and decrease the expression levels of proinflammatory cytokines [71, 72]. SCFAs promote the expression of HIF-1α through GPR41 and increase IL-22 production, thus protecting the gut from inflammation [24]. GPR43 is expressed on a variety of immune cells and maintains immune balance by inhibiting the production of inflammatory mediators, and GPR43 deficiency may lead to the exacerbation of DSS-induced colitis [73, 74]. GPR109A is an important mediator of the action of butyrate in the colon, promoting the expression of the anti-inflammatory cytokine IL-10 by regulating the function of antigen-presenting cells [75]. Our study revealed that *B. velezensis* MZ09 restored the levels of SCFAs and significantly increased the expression of SCFA receptors (GPR41/43/109A) (especially that of GPR43) in colon tissue. These findings indicated that *B. velezensis* MZ09 mitigated DSS-induced colitis in piglets via the SCFAs–GPRs axis.

As a key receptor for inflammation regulation, GPR43 has significant regulatory functions in colitis models [76]. Previous studies have demonstrated that SCFAs are capable of regulating the expression of signal transducers and STAT3 by triggering GPR43 activation [77]. The STAT3 signaling pathway maintains gut homeostasis by regulating processes such as apoptosis, the inflammatory response, and the immune response [78–80]. STAT3 signaling pathway activation requires phosphorylation, followed by dimerization, and then transportation through the nuclear membrane into the nucleus to achieve transcriptional regulation of target genes [81]. The STAT3/IL-10 signaling axis is of utmost importance in the restoration of the intestinal epithelium. Importantly, the absence of this signaling pathway is strongly associated with increased vulnerability to IBD [82, 83]. In the present study, in the MZ09 + DSS group, an increase in STAT3 phosphorylation levels was observed, and IL-10 expression was increased. These findings confirmed the mechanism by which *B. velezensis* MZ09 promoted the secretion of anti-inflammatory factors through the GPR43–STAT3 signaling axis. Notably, partial STAT3 phosphorylation was also observed in the challenge group, which was related to the increase in IL-6 levels induced by DSS. IL-6 activates STAT3 and increases its phosphorylation levels, which is related to STAT3 function in innate immunity [84, 85]. Previous studies have shown that HIF-1α expression is regulated by the STAT3 signaling pathway [86]. Intestinal mucosal hypoxia is a characteristic of the microenvironment in the pathology of IBD, and its degree is positively correlated with disease progression [87, 88]. In this study, *B. velezensis* MZ09 ameliorated the gut barrier damage induced by DSS through the upregulation of IL-10 and HIF-1α expression.

Research findings have indicated that the DSS-induced colitis model suppresses colonic epithelial cell proliferation and triggers cell apoptosis [89]. STAT3 activation enhances antiapoptotic signaling, whereas STAT3 deficiency exacerbates epithelial cell apoptosis [90–92]. Bcl-2 and Bax are the two most important proteins that regulate cell apoptosis. Bcl-2 prevents apoptosis, whereas Bax promotes apoptosis [93, 94]. Our study demonstrated that within the MZ09 + DSS group, Bcl-2 protein expression was notably upregulated, whereas Bax protein expression was significantly downregulated. In contrast, the DSS group exhibited the opposite pattern. This study revealed that *B. velezensis* MZ09 protected colonic cells from DSS-induced apoptosis. Research has shown that Bax promotes apoptosome formation by mediating the permeabilization of the mitochondrial outer membrane and then activates Caspase-3, an important protein for cell apoptosis [95, 96]. The results revealed that the activation level of Caspase-3 in the DSS group was markedly greater than that in the MZ09 + DSS group. Additionally, we examined transmission electron microscopy images of the colon. In the DSS group, the mitochondrial morphology significantly changed, including an increase in volume, swelling, and destruction of the cristae structure. However, in the MZ09 + DSS group, the mitochondrial damage caused by DSS was significantly alleviated. Some studies have shown that mitochondrial damage may lead to the downregulation of HIF-1α expression by disrupting the adaptive response of cells to hypoxia [97, 98]. *B. velezensis* MZ09 potentially regulates mitochondrial damage by activating STAT3 to promote HIF-1α. In addition, a recent study revealed that the endogenous mitochondrial pathway of apoptosis activates Caspase-3/GSDME to induce pyroptosis [99]. We hypothesized that DSS causes pyroptosis through mitochondrial damage. Our study also revealed that NLRP3 activation by HIF-1α was inhibited in the presence of mitochondrial damage [100]. The NLRP3 inflammasome has been recognized as a pivotal mechanism underlying intestinal inflammation in the DSS-induced colitis model. Upon NLRP3/GSDMD pathway activation, cells undergo rupture, thereby releasing proinflammatory factors such as IL-1β and IL-18. This release initiates the process of pyroptosis [101–103]. In our study, supplementation with *B. velezensis* MZ09 effectively suppressed the assembly of the NLRP3 inflammasome and the expression of its downstream effector molecules, IL-1β and IL-18. These findings indicate that *B. velezensis* MZ09 exerts a protective effect on the intestine by modulating mitochondria-related processes and pyroptosis pathways.

## Conclusions

This study confirmed that *Bacillus velezensis* MZ09 increases SCFA levels in the intestine through the BSCFA biosynthetic route. Its therapeutic action is predominantly accomplished by facilitating STAT3 signaling pathway activation via the GPR43 receptor. By mitigating DSS-induced mitochondrial damage and NLRP3 inflammasome-mediated epithelial cell pyroptosis, *Bacillus velezensis* MZ09 effectively strengthens intestinal barrier integrity. This study provides a solid theoretical basis for the use of acid-producing probiotics as functional feed additives in regulating intestinal health, suppressing inflammatory responses, and elucidating the immune regulatory mechanisms mediated by intestinal metabolic products.

## Supplementary Information

Additional file 1: Table S1. Composition and nutrient levels of basal diets. Table S2. DAI scoring rules. Table S3. Immunofluorescent antibody. Table S4. Elisa kit. Table S5. Primary antibodies. Fig. S1. Bacterial motility. Fig. S2. Images of pig colon morphology after slaughter. Fig. S3. Representative H&E-stained images of the pig colon.

## Data Availability

The original contributions presented in the study are included in the article, and further inquiries can be directed to the corresponding author.

## Abbreviations

Bax: BCL2-associated X
Bcl-2: B-cell lymphoma-2
BSCFAs: Branched-chain short-chain fatty acids
Caspase-1: Cysteinyl aspartate specific proteinase-1
Caspase-3: Cysteinyl aspartate specific proteinase-3
CD: Crohn’s disease
CFU: Colony-forming unit
DAI: Disease activity index
DAO: Diamine oxidase
D-LA: D-Lactic acid
DSS: Dextran sodium sulfate
ELISA: Enzyme-linked immunosorbent assay
GPRs: G protein-coupled receptors
HE: Histopathologic examination
HIF-1: Hypoxia-inducible factor 1
IBD: Inflammatory bowel disease
LB: Luria-Bertani
LPS: Lipopolysaccharide
NLRP3: Nucleotide-binding oligomerization domain, leucine- rich repeat and pyrin domain-containing 3
PCoA: Principal coordinates analysis
SCFAs: Short-chain fatty acids
STAT3: Signal transducer and activator of transcription 3
UC: Ulcerative colitis
