# Supplementary material for: Bacillus velezensis MZ09 alleviates DSS-induced colitis in piglets by remodeling the intestinal microbiota activating the SCFAs–GPR43–STAT3 pathway and suppressing NLRP3 inflammasome-mediated pyroptosis
Source: J Anim Sci Biotechnol. 2025 Aug 29;16:120. doi: 10.1186/s40104-025-01262-1 (PMC12395920; doi:10.1186/s40104-025-01262-1)
Supplement: Supplementary file 1 — Additional file 1: Table S1. Composition and nutrient levels of basal diets. Table S2. DAI scoring rules. Table S3. Immunofluorescent antibody. Table S4. Elisa kit. Table S5. Primary antibodies. Fig. S1. Bacterial motility. Fig. S2. Images of pig colon morphology after slaughter. Fig. S3. Representative H&E-stained images of the pig colon. [file 40104_2025_1262_MOESM1_ESM.docx]

**Table S1** Composition and nutrient levels of basal diets (as-fed basis%)

| Ingredients | Content | Nutrient levels | Content |
| --- | --- | --- | --- |
| Corn | 55.89 | Net Energy（Mcal/kg） | 2.69 |
| Soybean meal | 20.89 | Crude protein | 18.51 |
| Rice bran meal | 2.48 | Lysine | 1.23 |
| Whey powder | 2.98 | Methionine | 0.35 |
| Glucose | 1.99 | Threonine | 0.73 |
| Expanded soybean | 7.96 | Tryptophan | 0.25 |
| Fish meal | 1.99 | Calcium | 0.84 |
| Soybean oil | 1.99 | Total phosphorus | 0.68 |
| Calcium hydrogen phosphate | 0.99 | Available phosphorus | 0.44 |
| Limestone | 0.96 |  |  |
| NaCl | 0.4 |  |  |
| Choline chloride | 0.1 |  |  |
| L-Lysine | 0.32 |  |  |
| DL-Methionine | 0.02 |  |  |
| L-Threonine | 0.04 |  |  |
| Premix | 1.00 |  |  |
| Total | 100.00 |  |  |

^1^ Premix provided the following per kilogram of feed: vitamin A, 12,500 IU; vitamin D_3_, 2,800 IU; vitamin E, 30 IU; vitamin K_3_, 5 mg; vitamin B_1_, 1.5 mg; vitamin B_6_, 3 mg; vitamin B_12_, 40 μg; riboflavin, 15 mg; pantothenic acid, 15 mg; niacin, 40 mg; folic acid, 1 mg; biotin, 0.08 mg; Mn, 4 mg; Fe, 100 mg; Zn, 80 mg; Cu, 6 mg; I, 0.7 mg; Se, 0.48 mg

^2^ Crude protein were analyzed values, others were calculated

**Table S2** DAI scoring rules

| **Score** | **Percentage of Weight Loss** | **Diarrhoea Severity** | **Faecal Occult Blood Test** |
| --- | --- | --- | --- |
| 0 | 0% (No loss) | Normal stool | No purplish red colour within 2 minutes |
| 1 | 1-5% | Soft stool, formed | Gradual purple-red colouration within 1-2mins |
| 2 | 5-10% | Pasty, unformed | Fuchsia colour within 1min |
| 3 | 10-20% | Liquid, clear separation of faecal water | Fuchsia colour within 10s |
| 4 | >20% |  | Immediate fuchsia colour |

**Table S3** Immunofluorescent antibody.

| **Antibody** | **Cat. No.** | **Concentration** | **Supplier** |
| --- | --- | --- | --- |
| Caspase-3 | GB12532 | IHC-P: 1:500 | Servicebio, Hubei, China |
| MUC-2 | GB120002 | IHC-P: 1:500 | Servicebio, Hubei, China |
| Ki67 | GB121141 | IHC-P: 1:500 | Servicebio, Hubei, China |

**Table S4** Elisa kit

| **Elisa kit** | **Cat. No** | **Supplier** |
| --- | --- | --- |
| D-LA | HB468-Pg | Hnybio, Shanghai, China |
| DAO | HB281-Pg | Hnybio, Shanghai, China |
| LPS | HB022-Pg | Hnybio, Shanghai, China |

**Table S5** Primary antibodies

| **Antibody** | **Cat. No.** | **Concentration** | **Supplier** |
| --- | --- | --- | --- |
| β-actin | AC026 | WB: 1:10000 | ABclonal, Hubei, China |
| GPR41 | A12636 | WB: 1:1000 | ABclonal, Hubei, China |
| GPR43 | A18592 | WB: 1:1000 | ABclonal, Hubei, China |
| GPR109A | A15611 | WB: 1:1000 | ABclonal, Hubei, China |
| Occludin | A2601 | WB: 1:1000 | ABclonal, Hubei, China |
| Claudin1 | A11530 | WB: 1:1000 | ABclonal, Hubei, China |
| ZO-1 | A25306 | WB: 1:1000 | ABclonal, Hubei, China |
| Bax | A19684 | WB: 1:1000 | ABclonal, Hubei, China |
| IL-6 | WL02841 | WB: 1:1000 | Wanleibio, Shenyang, China |
| TNF-α | WL01581 | WB: 1:1000 | Wanleibio, Shenyang, China |
| STAT3 | WL01836 | WB: 1:500 | Wanleibio, Shenyang, China |
| p-STAT3 | WLP2412 | WB: 1:500 | Wanleibio, Shenyang, China |
| HIF-1α | WL01607 | WB: 1:500 | Wanleibio, Shenyang, China |
| IL-10 | WL03088 | WB: 1:1000 | Wanleibio, Shenyang, China |
| Bcl-2 | WL01556 | WB: 1:500 | Wanleibio, Shenyang, China |
| NLRP3 | WL02635 | WB: 1:1000 | Wanleibio, Shenyang, China |
| Caspase-1 | WL03450 | WB: 1:1000 | Wanleibio, Shenyang, China |
| GSDMD | WL05686 | WB: 1:500 | Wanleibio, Shenyang, China |
| IL-1β | WL00891 | WB: 1:500 | Wanleibio, Shenyang, China |
| IL-18 | WL01127 | WB: 1:1000 | Wanleibio, Shenyang, China |

**Fig. S1** Bacterial motility

**
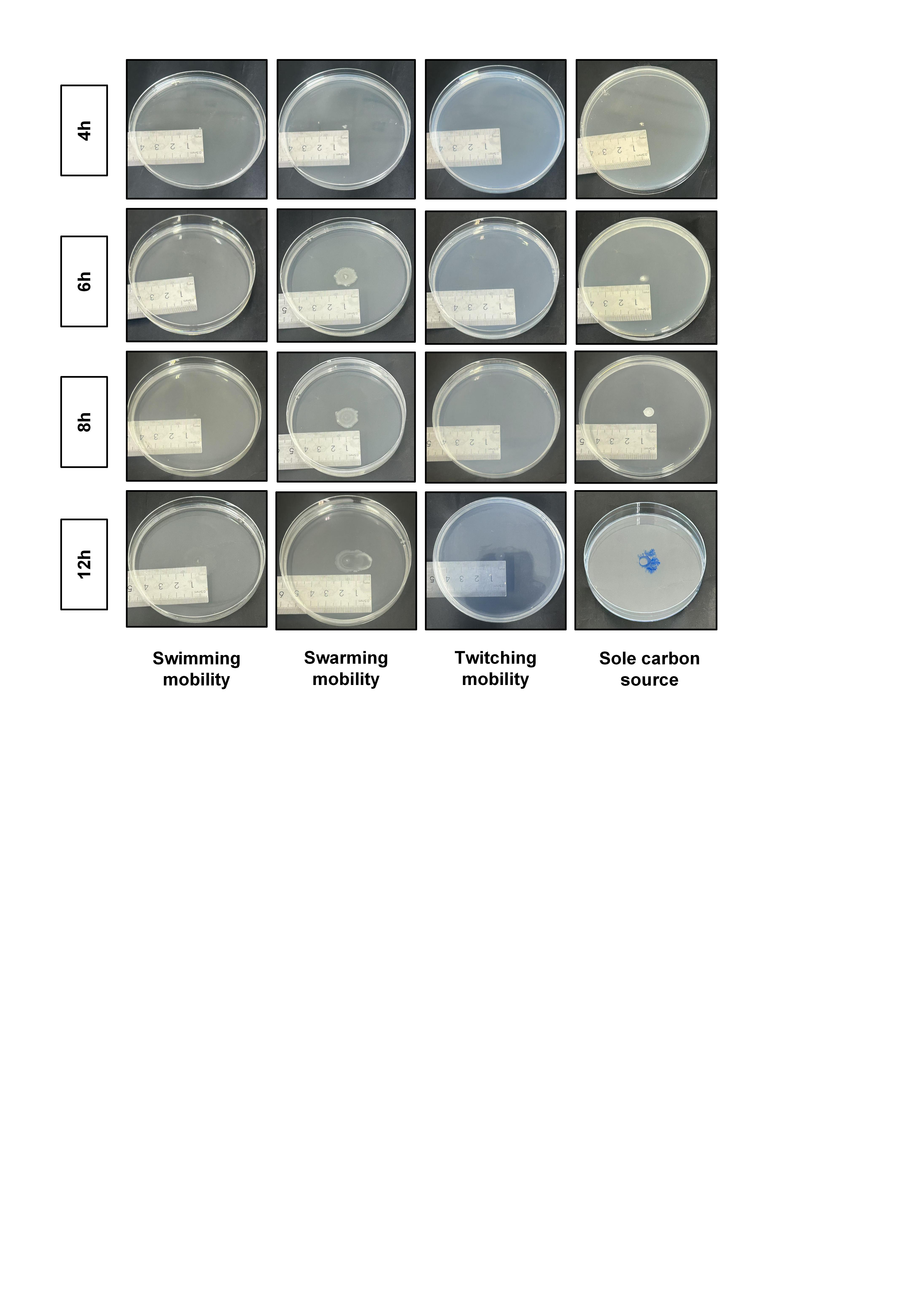
**

**Fig. S2** Images of pig colon morphology after slaughter

**
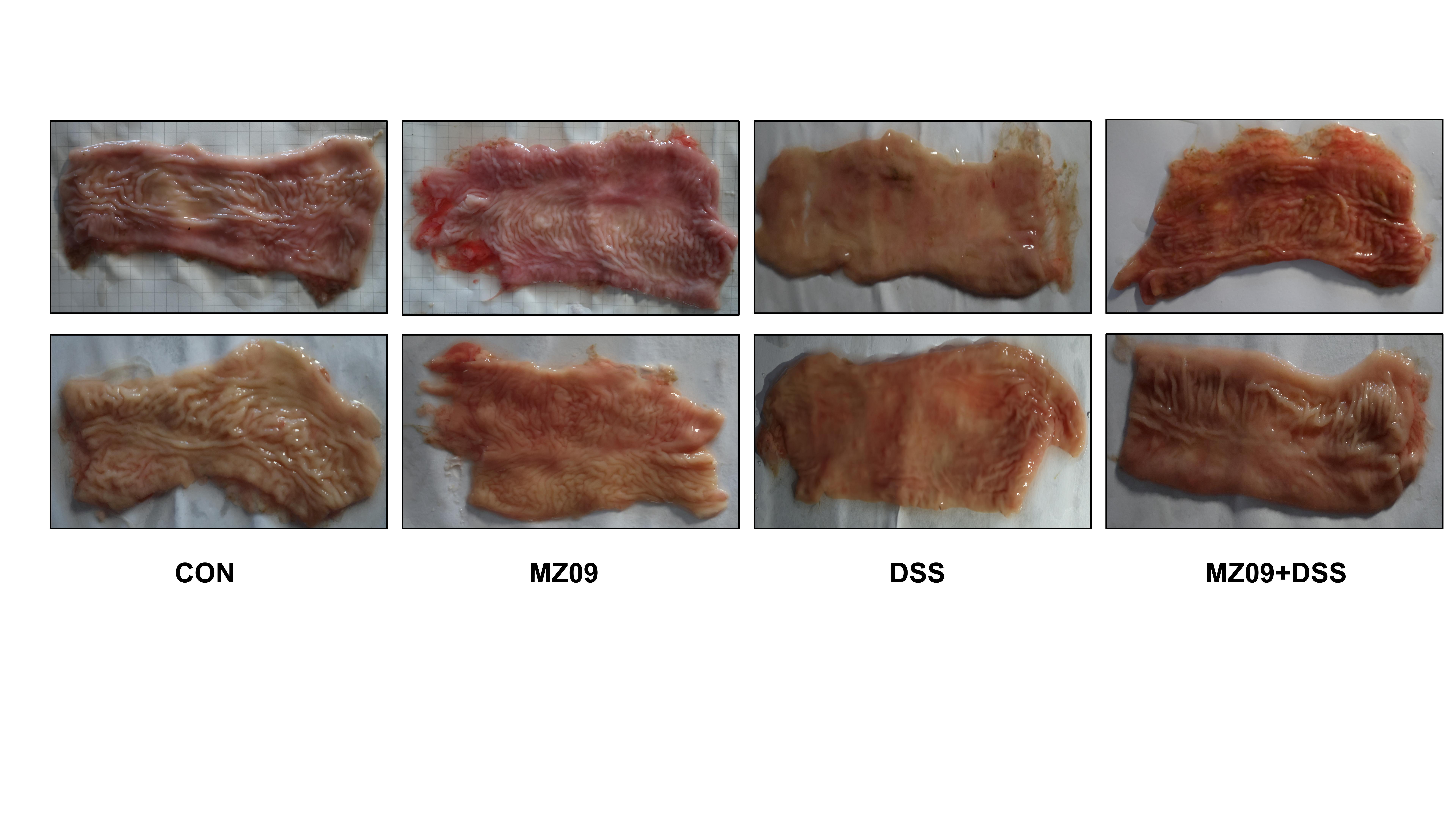
**

**Fig. S3** Representative H&E-stained images of the pig colon

**
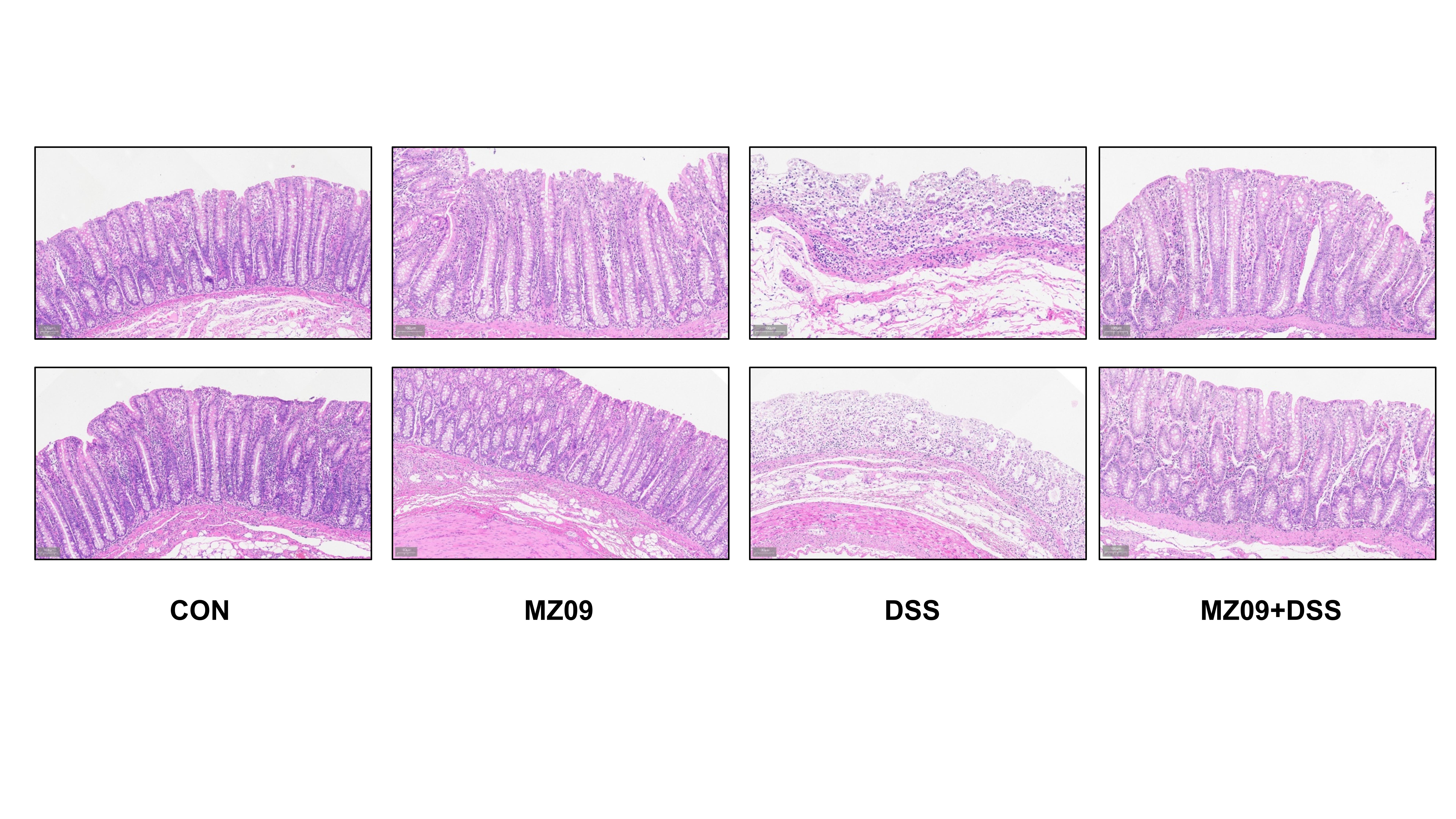
**
